# Exploring the Halogen-Bonded Cocrystallization Potential of a Metal-Organic Unit Derived from Copper(ii) Chloride and 4-Aminoacetophenone

**DOI:** 10.3390/ma13102385

**Published:** 2020-05-22

**Authors:** Vinko Nemec, Katarina Lisac, Marin Liović, Ivana Brekalo, Dominik Cinčić

**Affiliations:** Department of Chemistry, Faculty of Science, University of Zagreb, Horvatovac 102a, HR-10000 Zagreb, Croatia; vnemec@chem.pmf.hr (V.N.); katarina.lisac@chem.pmf.hr (K.L.); marin.liovic1@gmail.com (M.L.); ivana.brekalo2@gmail.com (I.B.)

**Keywords:** halogen bonding, hydrogen bonding, cocrystals, liquid assisted grinding, coordination compounds

## Abstract

In this work, we describe a novel halogen-bonded metal-organic cocrystal involving a square-planar Cu(ii) complex and 1,4-diiodotetrafluorobenzene (**14tfib**) by utilizing an amine ligand whose pendant acetyl group enables halogen bonding. The cocrystal was prepared by both mechanochemical synthesis (liquid-assisted grinding) and the conventional solution-based method. Crystal structure determination by single crystal X-ray diffraction revealed that the dominant supramolecular interactions are the I···O halogen bond between **14tfib** and CuCl_2_(**aap**)_2_ building blocks, and the N–H···Cl hydrogen bonds between CuCl_2_(**aap**)_2_ molecules. The combination of halogen and hydrogen bonding leads to the formation of a 2D network. Overall, this work showcases an example of the possibility for extending the complexity of metal-organic crystal structures by using halogen bonding in a way that does not affect other hydrogen bonding synthons.

## 1. Introduction

In the past three decades, the halogen bond has increasingly proven itself as a supramolecular interaction that can be used alongside the well-researched hydrogen bond in crystal engineering [1,2,3]. Key points that allow this are: (a) the halogen bond is an attractive interaction between a positive area of electrostatic potential on covalently bound halogen atoms (Br, I) and Lewis bases (nucleophilic atoms such as O, N, S, Se, P, etc.) that ranges from weak to very strong (10–150 kJ mol^−1^) [1,4,5], (b) it can be tuned by varying the donor atom used [6,7,8,9] or by changing its polarization [4,10] due to the fact that the halogen bond donor capacity results from the formation of a σ-hole, an electrophilic region, on the donor atom, (c) as a result, it is more directional than the hydrogen bond [1,4,6,11], and (d) it can form with acceptor atoms and species that are less receptive to hydrogen bonding [1,12,13]. These features of halogen bonding are especially interesting from the perspective of building larger acceptor molecules and more complex crystal structures, since they potentially provide a way to avoid synthon crossover that results from relying on only one interaction type [12,14]. 

While the majority of reports have focused on organic halogen-bonded crystals [1,2,3,10], the use of halogen bonding in the crystal engineering of metal–organic materials so far remains a challenge [15,16,17,18,19]. A number of reports and reviews have been published dealing with halogen bonding in single component metal–organic solids [15,16,17,20,21,22,23]. Most systematic studies of multicomponent metal–organic materials have focused on the utilization of halogen bonding with halide and pseudohalide (CN^−^, SCN^−^, OCN^−^, and similar) ligands and ions, as they have shown to be reliable in the synthesis of ionic metal–organic materials [24,25,26,27,28,29,30,31,32,33] and metal–organic cocrystals [15,34,35,36]. Several promising alternative approaches to obtaining neutral metal–organic materials have also recently been presented, generally focused on cocrystallizing neutral halogen bond donors with metal complex subunits that have either large chelating ligands (such as imines [37,38], acetylacetonates [39,40], or pyridine derivatives [41]) with pendant acceptor groups, or neutral monodentate ligands such as morpholine or thiomorpholine [42] coordinated to the metal complex. In all of the mentioned approaches an overwhelming majority of examples feature metal complex subunits that are chelated by bidentate (or tridentate) organic ligands. Searching the Cambridge Structural Database [43] for cocrystals of the selected iodoperfluorinated donors and metal–organic or organometallic species as halogen bond acceptors has resulted in a relatively small number of datasets (Table 1). The data obtained show that there are fewer cases where halogen bonds are formed with monodentate organic ligands than cases where they are formed with bidentate organic ligands, or directly bound halide or pseudohalide (CN^−^, SCN^−^, OCN^−^, and similar) ligands. This is presumably because metal complexes that have monodentate ligands are less stable in solution in comparison to chelate metal complexes containing bidentate ligands [44], so the decomposition of the metal complex in solution significantly hinders the desired cocrystallization of the complex subunits with the halogen bond donor.

Our interest in the halogen bonding of coordination compounds is in the design and synthesis of extended structures, cocrystals, consisting of metal–organic complexes connected by halogen bonds. In our previous work, we have established that the peripherally located carbonyl functional group can function as a good halogen bond acceptor, allowing us to tune the properties of the material either via changing the metal center or the linking donor molecule [37,38]. Therein, we used a bidentate imine ligand in order to minimize or avoid the possibility of ligand detachment and separate cocrystallization with the donor molecules.

**Figure 1:**
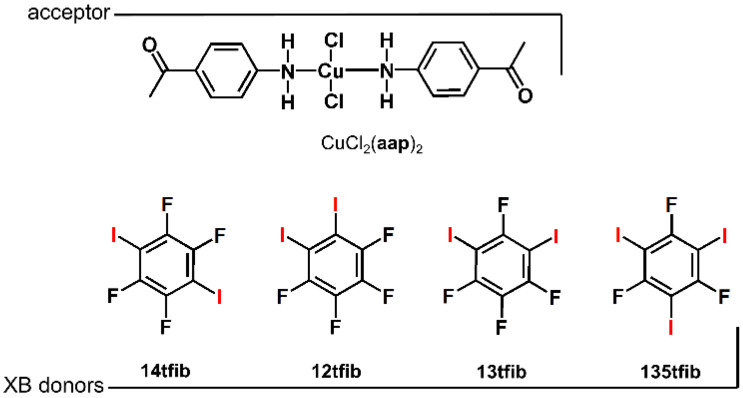
The halogen bond acceptor used in this study, a coordination compound derived from copper(ii) chloride and 4-aminoacetophenone, and halogen bond donors, perhalogenated benzenes.

In this work, we were interested in testing the possibility of obtaining halogen-bonded cocrystals with coordination compounds featuring monodentate ligands. Additionally, we wanted to observe whether chloride ligands would hinder the formation of the desired halogen bond. To explore this, we have prepared a square-planar copper(ii) complex that has both two chloride ligands and two 4-aminoacetophenone (**aap**) molecules as monodentate ligands, whose pendant acetyl group enables halogen bonding. For the cocrystal synthesis as halogen bond donors, we have selected a series of perhalogenated benzenes that differ in the number and positions of donor atoms: 1,4-diiodotetrafluorobenzene (**14tfib**), 1,3-diiodotetrafluorobenzene (**13tfib**), 1,2-diiodotetrafluorobenzene (**12tfib**), and 1,3,5-triiodotrifluorobenzene (**135tfib**) (Figure 1).

## 2. Materials and Methods 

### 2.1. Synthesis of CuCl_2_(**aap**)_2_

Copper(ii) chloride dihydrate (1.00 g, 5.88 mmol) and **aap** (1.58 g, 11.8 mmol) were mixed with 30.0 mL of methanol in a round-bottom flask and stirred while heating under reflux for 1.0 h. The obtained crystals were filtered and washed with acetone.

### 2.2. Mechanochemical Experiments

In order to explore the cocrystallization of CuCl_2_(**aap**)_2_ with selected halogen bond donors, we performed mechanochemical experiments by liquid-assisted grinding (LAG) [45,46] of the reactants in a 1:1 donor : complex stoichiometric ratio, and in the presence of a small amount of acetonitrile (see ESI, Table S1). Milling was conducted under normal laboratory conditions (temperature ca. 25 °C, 40–60% relative humidity) for 30 min in a Retsch MM200 Shaker Mill (Retsch GmbH, Haan, Germany) operating at 25 Hz frequency, using a 10 mL plexiglass jar and two stainless steel balls 7 mm in diameter. All reactants and products were characterized by powder X-ray diffraction (PXRD).

The [CuCl_2_(**aap**)_2_](**14tfib**) cocrystal was prepared by milling a mixture of 99.6 mg (0.246 mmol) CuCl_2_(**aap**)_2_ and 100.0 mg (0.248 mmol) **14tfib** for 30 min along with 20.0 μL of ethanol.

### 2.3. Crystallization Experiments

A CuCl_2_(**aap**)_2_ single crystal was obtained by slow evaporation at room temperature of the solution obtained by reflux synthesis. The [CuCl_2_(**aap**)_2_](**14tfib**) single crystal was obtained after dissolving a mixture of CuCl_2_(**aap**)_2_ (50.0 mg, 0.123 mmol) and **14tfib** (50.0 mg, 0.124 mmol) in 5.0 mL of a hot mixture of tetrahydrofuran and ethanol (volume ratio 1:2) and the subsequent cooling and solvent evaporation at room temperature for two days.

### 2.4. Thermal Analysis

Thermal measurements were performed on a Mettler–Toledo TGA/DSC 3^+^ module (Mettler-Toledo, Greifensee, Switzerland). Samples were placed in open 70 µL alumina pans and heated from 25 to 600 °C at a rate of 10 °C min^−1^ under an oxygen flow of 50 mL min^−1^. The data collection and analysis were performed using the program package STARe Software v15.00 (Mettler-Toledo GmbH, Giessen, Germany) [47].

### 2.5. Single-Crystal X-ray Diffraction Experiments

The crystal and molecular structures of the prepared samples were determined by single crystal X-ray diffraction. The details of the data collection and crystal structure refinement are listed in Table S2. The diffraction data for CuCl_2_(**aap**)_2_ and [CuCl_2_(**aap**)_2_](**14tfib**) were collected at 295 K. Diffraction measurements were made on an Oxford Diffraction Xcalibur Kappa CCD X-ray diffractometer with graphite-monochromated MoKα (*λ* = 0.71073 Å) radiation. The datasets were collected using the ω scan mode over the 2*θ* range up to 54°. The programs CrysAlis CCD and CrysAlis RED (Oxford Diffraction Ltd., Abingdon, UK) were employed for data collection, cell refinement, and data reduction [48,49]. The structures were solved by direct methods and refined using the SHELXS, SHELXT, and SHELXL programs, respectively [50,51]. Structural refinement was performed on F^2^ using all data. Hydrogen atoms not involved in hydrogen bonding were placed in calculated positions and treated as riding on their parent atoms [*d*(C–H) = 0.93 Å and *U*_iso_(H) = 1.2 *U*_eq_(C)], while the others were located from the electron difference map. Parameters of the supramolecular interactions that are present in the prepared compounds are listed in Table S3. All calculations were performed using the WinGX crystallographic suite of programs [52]. The images of the molecular structures of compounds and their molecular packing projections were prepared by Mercury [53].

### 2.6. Powder X-ray Diffraction Experiments

PXRD experiments on the samples were performed on a PHILIPS PW 1840 X-ray diffractometer (Philips Analytical, Almelo, The Netherlands) with CuKα1 (1.54056 Å) radiation at 40 mA and 40 kV. The scattered intensities were measured with a scintillation counter. The angular range was from 3 to 40° (2*θ*) with steps of 0.02–0.03°, and the measuring time was 0.2–0.5 s per step. Data collection and analysis was performed using the program package Philips X’Pert (Philips Analytical, Almelo, The Netherlands) [54,55,56].

## 3. Results and Discussion

**Figure 2:**
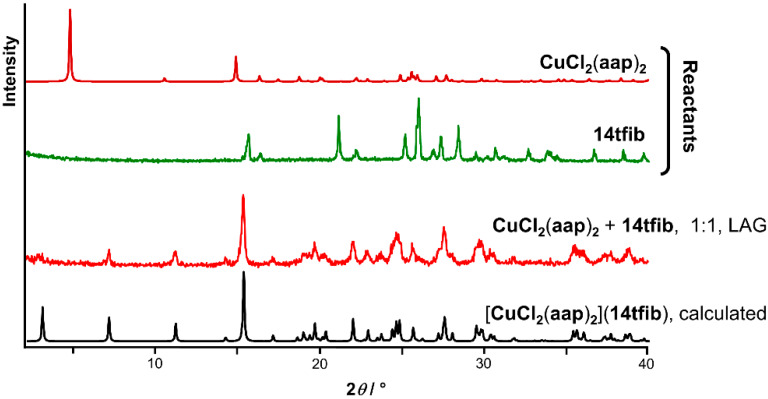
A comparison of the powder X-ray diffraction patterns of the reactants, the LAG product, and the pattern calculated from the [CuCl_2_(**aap**)_2_](**14tfib**) single crystal X-ray diffraction data.

The CuCl_2_(**aap**)_2_ complex was synthesized by reacting copper(ii) chloride dihydrate and the **aap** ligand. The structure was confirmed by X-ray diffraction on crystals grown from the solution obtained by reflux synthesis (see ESI, Figure S5). Our screening for cocrystal formation of the obtained complex was based on mechanochemical liquid-assisted grinding followed by PXRD analysis. Only one out of a total of four reactant combinations resulted in the formation of new crystalline products, as evidenced by the appearance of new Bragg reflections in the PXRD patterns upon milling (see ESI, Figure S1). A new product is formed by milling CuCl_2_(**aap**)_2_ with **14tfib** in the presence of a small amount (40.0 μL) of acetonitrile (Figure 2). In the other cases (see ESI, Figures S2–S4), the PXRD pattern obtained shows either a reactant mixture (milling with **135tfib**), a reactant mixture that includes the appearance of several new Bragg peaks (milling with **12tfib** in both stoichiometric ratios), or peaks of CuCl_2_(**aap**)_2_ (milling with **13tfib**). The LAG experiment with CuCl_2_(**aap**)_2_ and **14tfib** was accompanied by crystallization from an ethanolic solution of the reagents which resulted in single crystals suitable for X-ray diffraction. Given our previous experiences in cocrystallization from solutions of metal complexes that have monodentate ligands, it was quite astonishing that we were able to obtain crystals of the desired product, the [CuCl_2_(**aap**)_2_](**14tfib**) cocrystal. For example, our previous attempt to cocrystallize **14tfib** with a similar Cu(ii) complex derived from copper(ii) chloride and 4-nitroaniline resulted in the crystallization of a cocrystal of free 4-nitroaniline and **14tfib** [57]. The measured PXRD pattern of the new mechanochemical product was found to be in good agreement with the patterns calculated from the single crystal X-ray diffraction of [CuCl_2_(**aap**)_2_](**14tfib**) (Figure 2), showing that the mechanochemically prepared cocrystal was obtained as a pure single phase. Similar experiments for the potential cocrystallization of CuCl_2_(**aap**)_2_ and **12tfib** from the reagent solution yielded only separate reactant phases.

**Figure 3:**
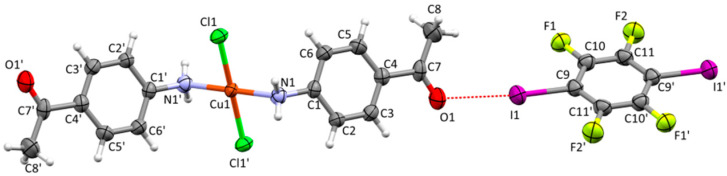
Molecular structure of [CuCl_2_(**aap**)_2_](**14tfib**) showing the atom-labelling scheme. Displacement ellipsoids are drawn at a 50% probability level, and H atoms are shown as small spheres of arbitrary radius (See ESI, Table S4, for the symmetry codes of symmetry equivalent atoms marked with an ′ symbol).

The molecular and crystal structure determination revealed that the asymmetric unit of [CuCl_2_(**aap**)_2_](**14tfib**) contains one half of a CuCl_2_(**aap**)_2_ molecule and one half of a **14tfib** molecule (Figure 3). The molecular structure of the CuCl_2_(**aap**)_2_ unit is in good agreement with that in the parent complex, with a root mean square deviation value of 0.1418 (see ESI, Figure S6). The central Cu(ii) atom has a square planar coordination geometry and is coordinated by two N atoms from two **aap** molecules and two Cl atoms, forming a structure with a square planar geometry (*d*(Cu1–N1) = 2.021 Å, *d*(Cu1–Cl1) = 2.251 Å, ∠(N1–Cu1–Cl1) = 88.8° and 91.2°, *τ*_4_ = *τ*_4_′ = 0) [58,59].

**Figure 4:**
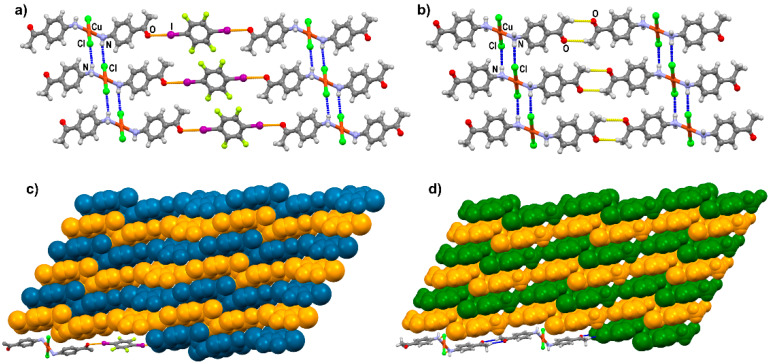
Parts of the crystal structure of (**a**) [CuCl_2_(**aap**)_2_](**14tfib**) showcasing the 2D network formed by a combination of halogen (colored orange) and hydrogen bonds (colored blue), (**b**) CuCl_2_(**aap**)_2_ with the 2D network resulting from a combination of N–H···Cl and C–H···O hydrogen bonds (colored blue and yellow, respectively). Layer stacking in (**c**) [CuCl_2_(**aap**)_2_](**14tfib**) and (**d**) CuCl_2_(**aap**)_2_. For clarity, adjacent layers are color-coded orange and blue/green.

In the crystal structure, each CuCl_2_(**aap**)_2_ unit is associated with two **14tfib** molecules via almost linear I···O halogen bonds (*d*(I1···O1) = 2.988 Å, ∠ (C1–I9···O1) = 173°), forming halogen-bonded chains. (Figure 4a). This halogen bond can be considered relatively strong, as evidenced not only by its linearity, but also by the relatively large value of the donor···acceptor distance shortening, 14.6%, with respect to the sum of the corresponding van der Waals radii of atoms [60]. Each coordinated Cl atom is only involved as an acceptor in N–H···Cl hydrogen bonding (*d*(N1···Cl1) = 3.466 Å, ∠ (N1–H1A···Cl1) = 165°) with the amino group of an adjacent molecule, similar to the CuCl_2_(**aap**)_2_ parent complex (Figure 4b). We therefore conclude that there appears to be no competition between the chloride ligand and the carbonyl functional group for halogen bonding with **14tfib**. The combination of halogen and hydrogen bonding leads to the formation of layers (Figure 4a). The overall structure results from the stacking of such layers along the [3 0 13] crystallographic direction (Figure 4c). 

Importantly, the CuCl_2_(**aap**)_2_ parent complex also forms supramolecular chains, where the CuCl_2_(**aap**)_2_ units—instead of being bridged by halogen bonding with **14tfib** molecules—are directly bound into a chain through pairs of C–H···O hydrogen bonds forming $R_{2}^{2}\left(8\right)$ motifs (*d* (C8···O1) = 3.61 Å, ∠ (C8–H8C···O1) = 168.0°) (Figure 4b). These supramolecular chains are again connected into a layer by a combination of N–H···Cl hydrogen bonds (*d* (N1···Cl1) = 3.474 Å, ∠ (N1–H2N···Cl1) = 171°). The final crystal structure is built by further stacking of the layers along the [5 0 14] crystallographic direction (Figure 4d). We hypothesize that this similarity between the crystal structures of the parent complex and the halogen-bonded cocrystal is the reason why **14tfib** easily forms a cocrystal with CuCl_2_(**aap**)_2_ in solution and mechanochemically, while other halogen bond donors of similar size and donor strength do not.

The thermal analysis by TGA reveals that the parent coordination compound and its halogen-bonded cocrystal decompose upon heating in two steps. Evaluating the inflection point temperature for the first step of thermal degradation (see ESI, Figures S7 and S8) reveals that both compounds have similar thermal stabilities, 137 °C for CuCl_2_(**aap**)_2_ and 145 °C for [CuCl_2_(**aap**)_2_](**14tfib**). The similarity in thermal degradation temperature is interesting, considering their different compositions and supramolecular architectures, as well as the fact that the pure halogen bond donor, **14tfib**, has a melting point of ~108 °C.

## 4. Conclusions

To conclude, we have successfully obtained a cocrystal with a one-dimensional halogen-bonded metal–organic architecture, using a copper(ii) coordination compound that has a monodentate ligand with peripherally located carbonyl oxygen atoms. As in our previous work, the carbonyl oxygen of the acetyl group has proven its potential as a good halogen bond acceptor, although the present study indicates that its potential can be severely limited by other effects. In the case of the CuCl_2_(**aap**)_2_ complex, a molecule with a linear distribution of donor atoms (such as **14tfib**) can replace the C–H···O hydrogen bonding motif of the parent complex by insertion. Other molecules of similar size and donor strength, but with donor atoms at an angle, like **12tfib**, **13tfib**, and **135tfib**, cannot because their incorporation would require a significant alteration of the overall crystal structure and connectivity. Disrupting the strong N–H···Cl hydrogen bond motif connecting the supramolecular chains would be especially difficult and severely limits the number of acceptable donor molecules. Furthermore, the strong N–H···Cl hydrogen bond motif effectively ‘masks’ the chloride ligand, preventing it from competing with the acetyl group for halogen bond formation. To overcome these limitations and further explore the competitiveness of halides with peripherally located ligands for halogen bonding, our future research will focus on systems with hydrogen bond-donating functional groups that are weaker than the amino group.

## Figures and Tables

**Table 1:** The number of unique datasets in the Cambridge Structural Database (version 5.41, March 2020) corresponding to cocrystals of coordination compounds with the selected series of halogen bond donors [43].

Donor	Monodentate Ligand	Bidentate Ligand	Halide and Pseudohalide
1,4-diiodotetrafluorobenzene	8 hits	18 hits	20 hits
1,3-diiodotetrafluorobenzene	no hits	2 hits	2 hits
1,2-diiodotetrafluorobenzene	no hits	no hits	3 hits
1,3,5-triiodotrifluorobenzene	no hits	5 hits	2 hits

## Supplementary Materials

The following are available online at https://www.mdpi.com/1996-1944/13/10/2385/s1, Figure S1: PXRD patterns of (a) CuCl_2_(**aap**)_2_, (b) **14tfib**, (c) product obtained by grinding CuCl_2_(**aap**)_2_ and **14tfib** in a 1:1 stoichiometric ratio, (d) calculated pattern from [CuCl_2_(**aap**)_2_](**14tfib**) single crystal data, Figure S2: PXRD patterns of (a) CuCl_2_(**aap**)_2_, (b) **12tfib**, (c) product obtained by grinding CuCl_2_(**aap**)_2_ and **12tfib** in a 1:2 stoichiometric ratio, (d) product obtained by grinding CuCl_2_(**aap**)_2_ and **12tfib** in a 1:1 stoichiometric ratio. Stars denote small peaks not belonging to either reactant, Figure S3: PXRD patterns of (a) CuCl_2_(**aap**)_2_, (b) product obtained by grinding CuCl_2_(**aap**)_2_ and **13tfib** in a 1:1 stoichiometric ratio, Figure S4: PXRD patterns of (a) **135tfib**, (b) CuCl_2_(**aap**)_2_, (c) product obtained by grinding CuCl_2_(**aap**)_2_ and **135tfib** in a 1:1 stoichiometric ratio, Figure S5: Molecular structure of CuCl_2_(**aap**)_2_ showing the atom-labelling scheme. Displacement ellipsoids are drawn at the 50% probability level, and H atoms are shown as small spheres of arbitrary radius. Symmetry codes of symmetry equivalent atoms marked with an ′ symbol are listed in Table S4, Figure S6: Molecular overlay of the metal complex molecule obtained from CuCl_2_(**aap**)_2_ data (colored red) with the metal complex molecule obtained from [CuCl_2_(**aap**)_2_](**14tfib**) data (colored blue). The metal complex structures are in good agreement with a root mean square deviation (RMSD) value of 0.1418 and a maximum distance of 0.2619, Figure S7: TG and DSC curves of CuCl_2_(**aap**)_2_, Figure S8: TG and DSC curves of [CuCl_2_(**aap**)_2_](**14tfib**), Table S1: Mechanochemical synthesis parameters, Table S2: Crystal data and refinement details for the prepared compounds, Table S3: Parameters of the supramolecular interactions and corresponding symmetry operators present in the prepared compounds, Table S4: Atom list and symmetry codes of symmetry equivalent atoms (marked with an ′ symbol) in the prepared compounds. CCDC 1997629 and 1997630 contain crystallographic data for this paper. These data can be obtained free of charge from the Director, CCDC, 12 Union Road, Cambridge, CBZ 1EZ, UK (Fax: +44-1223-336033; email: deposit@ccdc.cam.ac.uk or http://www.ccdc.cam.ac.uk).
