# Supplementary material for: Exploring the Halogen-Bonded Cocrystallization Potential of a Metal-Organic Unit Derived from Copper(ii) Chloride and 4-Aminoacetophenone
Source: Materials (Basel). 2020 May 22;13(10):2385. doi: 10.3390/ma13102385 (PMC7288111; doi:10.3390/ma13102385)
Supplement: Supplementary file 1 [file materials-13-02385-s001.pdf]

*materials*

MDPI

# SUPPORTING INFORMATION

*Communication*

# Exploring the Halogen-Bonded Cocrystallization Potential of a Metal-Organic Unit Derived from Copper(II) Chloride and 4-Aminoacetophenone

**Vinko Nemec, Katarina Lisac, Marin Liović, Ivana Brekalo and Dominik Cinčić ***

Department of Chemistry, Faculty of Science, University of Zagreb, Horvatovac 102a, HR-10000 Zagreb, Croatia; vnemec@chem.pmf.hr (V.N.); katarina.lisac@chem.pmf.hr (K.L.); marin.liovic1@gmail.com (M.L.); ivana.brekalo2@gmail.com (I.B.)

* Correspondence: dominik@chem.pmf.hr; Tel.: +385-1460-6362

Received: 22 April 2020; Accepted: 18 May 2020; Published: date

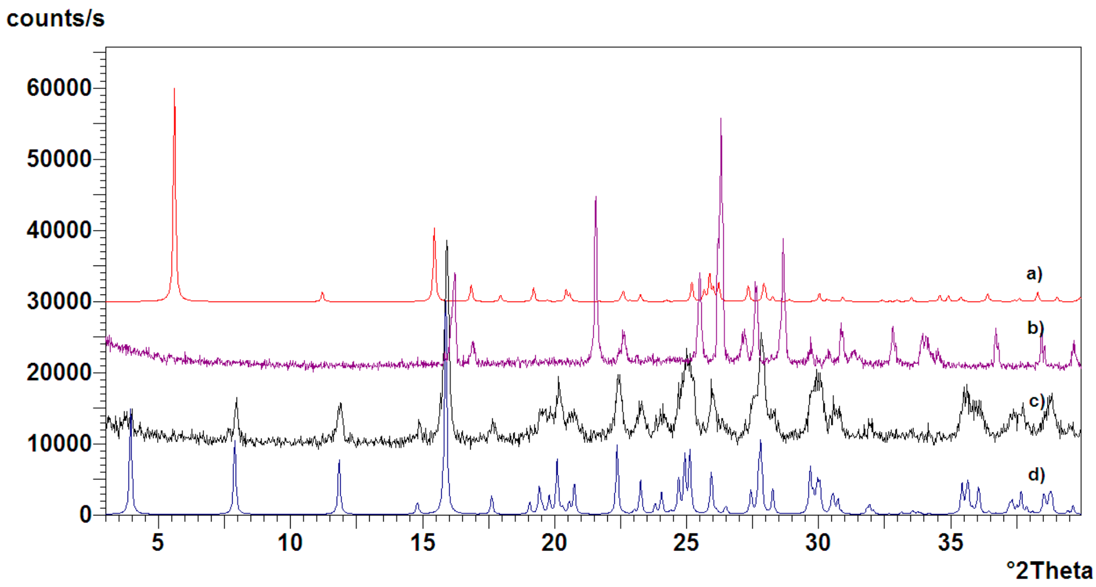


**Figure S1.** PXRD patterns of **a**) $CuCl_2$(**aap**)$_2$, **b**) **14tfib**, **c**) product obtained by grinding $CuCl_2$(**aap**)$_2$ and **14tfib** in a 1:1 stoichiometric ratio, d) calculated pattern from [$CuCl_2$(**aap**)$_2$](**14tfib**) single crystal data.

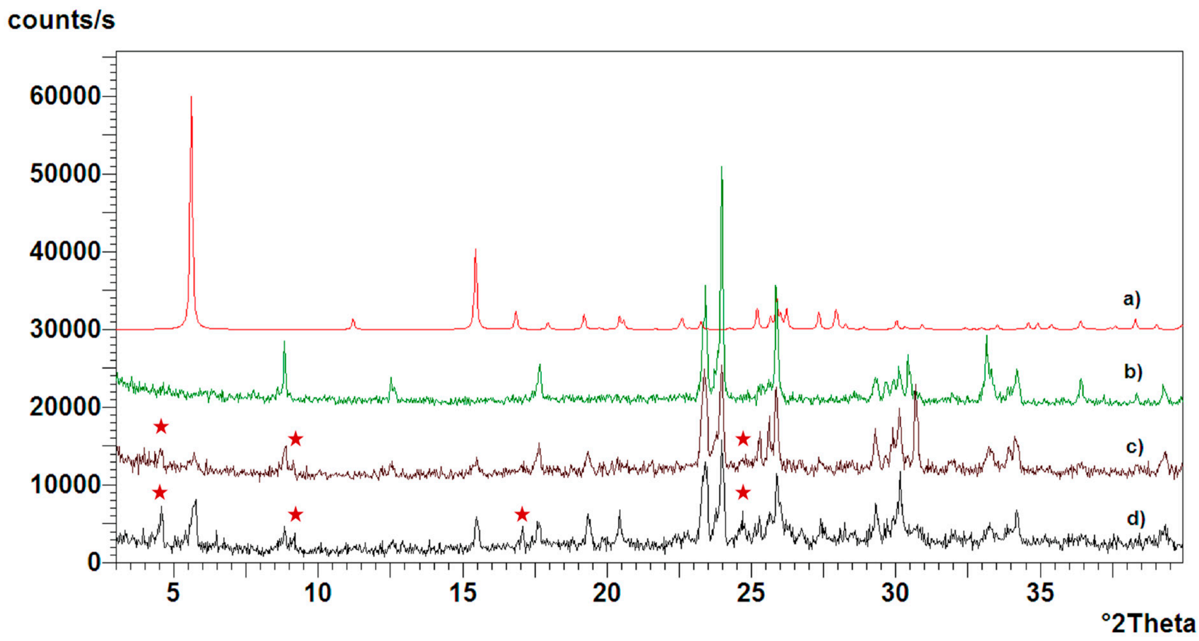


**Figure S2.** PXRD patterns of **a**) $CuCl_2$(**aap**)$_2$, **b**) **12tfib**, **c**) product obtained by grinding $CuCl_2$(**aap**)$_2$ and **12tfib** in a 1:2 stoichiometric ratio, d) product obtained by grinding $CuCl_2$(**aap**)$_2$ and **12tfib** in a 1:1 stoichiometric ratio. Stars denote small peaks not belonging to either reactant.

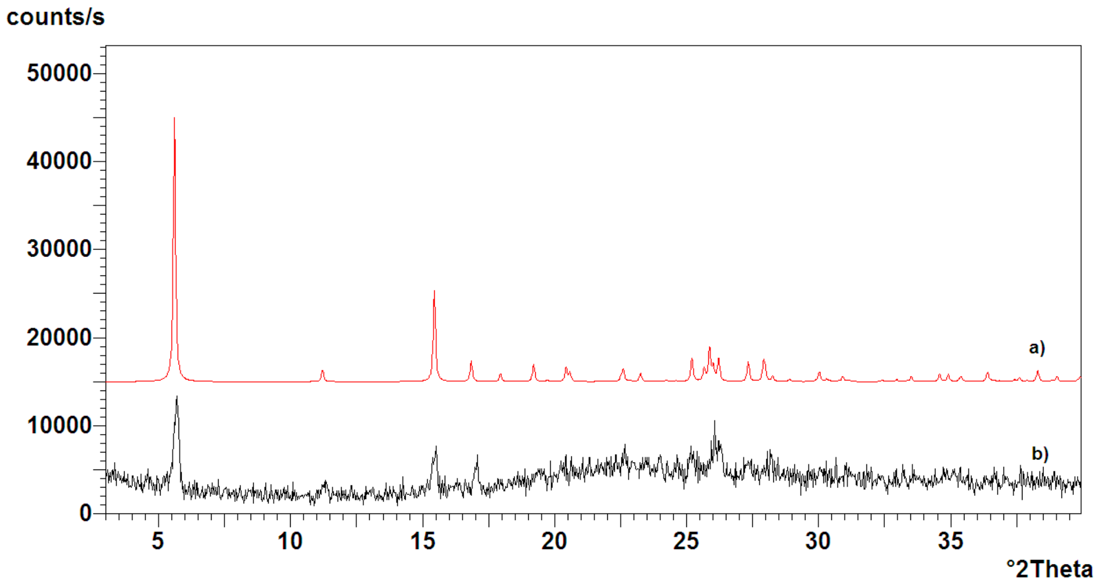


**Figure S3.** PXRD patterns of **a**) $CuCl_2$(**aap**)$_2$, **b**) product obtained by grinding $CuCl_2$(**aap**)$_2$ and **13tfib** in a 1:1 stoichiometric ratio. The other reagent (**13tfib**) is a liquid, so its PXRD pattern is omitted.

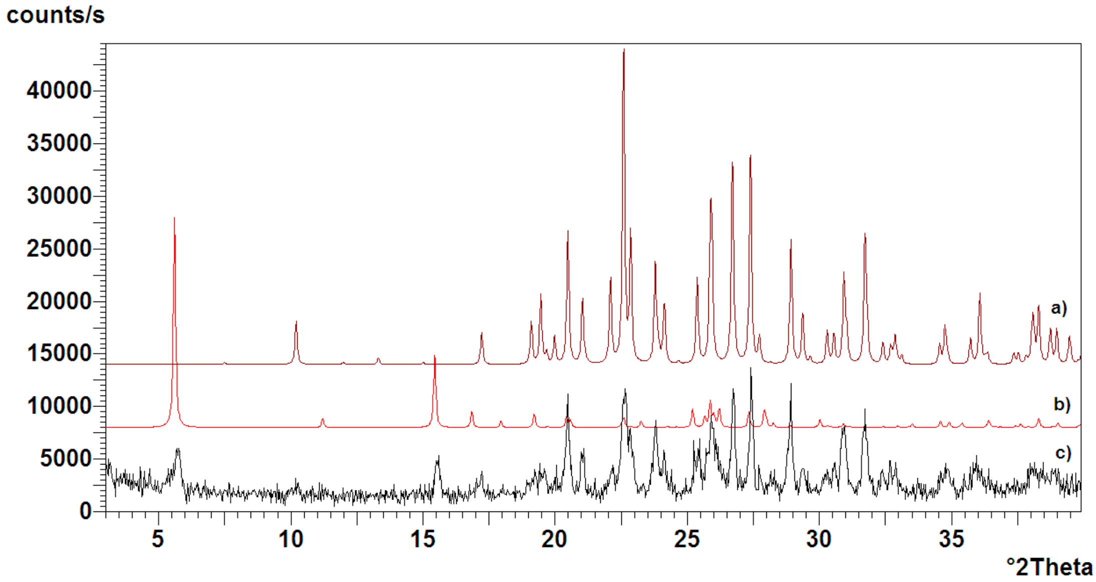


**Figure S4.** PXRD patterns of **a**) **135tfib**, **b**) $CuCl_2$(**aap**)$_2$, **c**) product obtained by grinding $CuCl_2$(**aap**)$_2$ and **135tfib** in a 1:1 stoichiometric ratio.

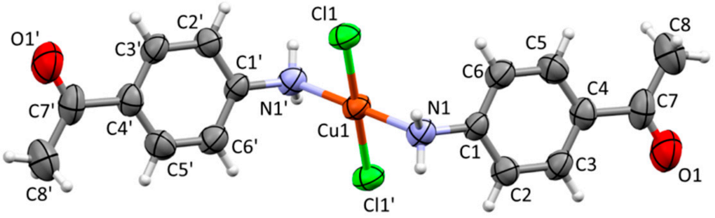


**Figure S5.** Molecular structure of $CuCl_2$(**aap**)$_2$ showing the atom-labelling scheme. Displacement ellipsoids are drawn at the 50 % probability level, and H atoms are shown as small spheres of arbitrary radius. Symmetry codes of symmetry equivalent atoms marked with an ' symbol are listed in Table S4.

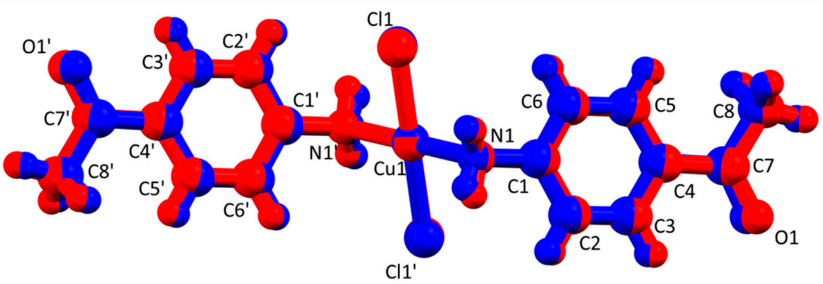


**Figure S6.** Molecular overlay of the metal complex molecule obtained from $CuCl_2$(**aap**)$_2$ data (colored red) with the metal complex molecule obtained from [$CuCl_2$(**aap**)$_2$](**14tfib**) data (colored blue). The metal complex structures are in good agreement with a root mean square deviation (RMSD) value of 0.1418 and a maximum distance of 0.2619.

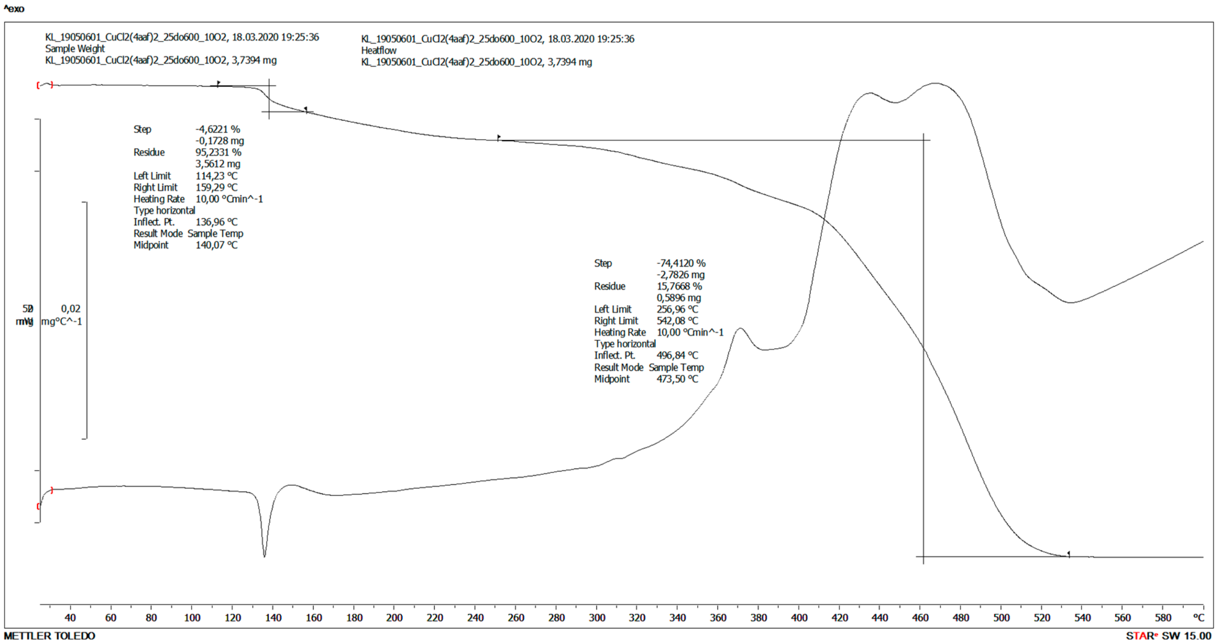


**Figure S7.** TG and DSC curves of CuCl2(**aap**)2.

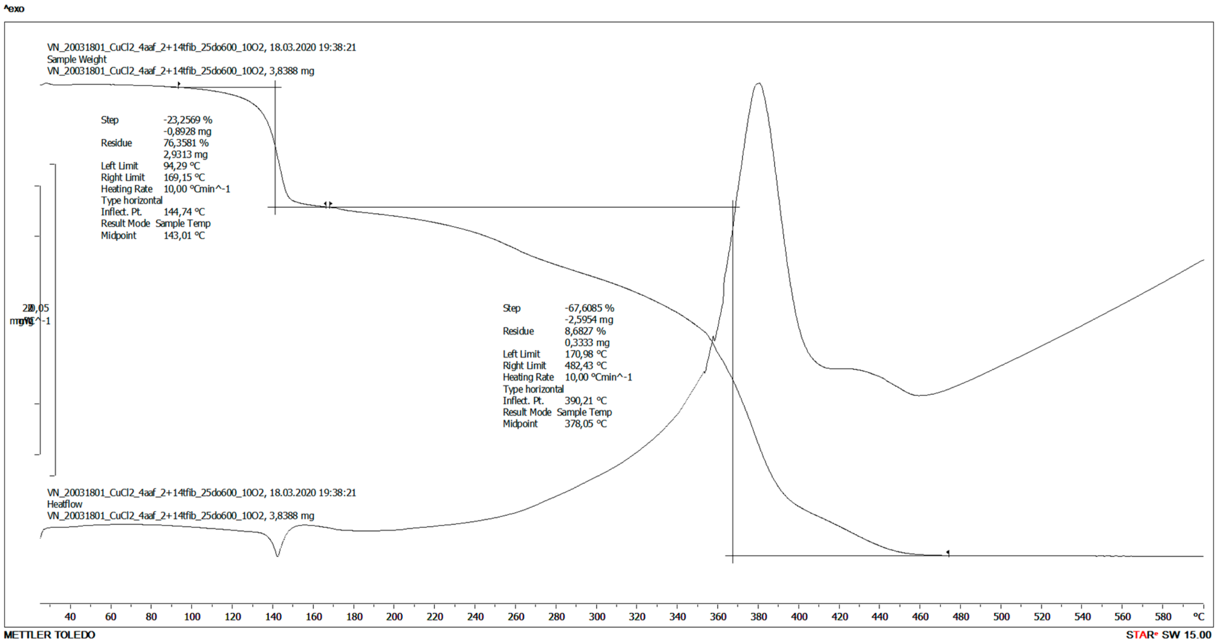


**Figure S8.** TG and DSC curves of [CuCl2(**aap**)2](**14tfib**).

**Table S1.** Mechanochemical Synthesis Parameters.

| $t_{milling}$ = 30 min, $\nu_{milling}$ = 25 Hz. | | | | |
|---|---|---|---|---|
| **Reactants** | **Mole Ratio** | ***m*(CuCl2(aap)2) / mg** | ***m* or *V*(donor)** | **Liquid** |
| CuCl2(**aap**)2 : **14tfib** | 1 : 1 | 99.6 | 100.0 mg | 40.0 µL acetonitrile |
| CuCl2(**aap**)2 : **12tfib** | 1 : 2 | 67.0 | 133.0 mg | 40.0 µL acetonitrile |
| | 1 : 1 | 100.0 | 99.3 mg | 40.0 µL acetonitrile |
| CuCl2(**aap**)2 : **13tfib** | 1 : 1 | 100.0 | 37.5 µL | 10.0 µL acetonitrile |
| CuCl2(**aap**)2 : **135tfib** | 1 : 1 | 100.0 | 125.9 mg | 40.0 µL acetonitrile |

**Table S2.** Crystal data and refinement details for the prepared compounds.

| | **$CuCl_2(aap)_2$** | **$[CuCl_2(aap)_2](14tfib)$** |
|---|---|---|
| Molecular formula | $C_{16}H_{18}CuCl_2N_2O_2$ | $(C_{16}H_{18}CuCl_2N_2O_2)(C_6F_4I_2)$ |
| $M_r$ | 404.76 | 806.62 |
| Crystal system | triclinic | triclinic |
| Space group | $P\bar{1}$ | $P\bar{1}$ |
| Crystal data: | | |
| $a$ / Å | 4.5094(12) | 4.6532(3) |
| $b$ / Å | 6.0113(14) | 6.0374(3) |
| $c$ / Å | 15.666(4) | 22.5789(12) |
| $\alpha$ / ° | 93.87(2) | 82.905(4) |
| $\beta$ / ° | 90.18(2) | 89.166(4) |
| $\gamma$ / ° | 92.38(2) | 89.192(4) |
| $V$ / Å$^3$ | 423.31(19) | 629.34(6) |
| $Z$ | 1 | 1 |
| $D_{calc}$ / g cm$^{-3}$ | 1.588 | 2.928 |
| $\lambda$(Mo$K_\alpha$) / Å | 0.71073 | 0.71073 |
| $T$ / K | 295 | 295 |
| Crystal size / mm$^3$ | 0.35 x 0.26 x 0.03 | 0.60 x 0.40 x 0.07 |
| $\mu$ / mm$^{-1}$ | 1.615 | 3.586 |
| $F$(000) | 207 | 385 |
| Refl. collected/unique | 2885 / 1481 | 8303 / 2400 |
| Parameters/restraints | 113/0 | 169/0 |
| $\Delta\rho_{max}$ , $\Delta\rho_{min}$ / e Å$^{-3}$ | 1.090; −1.135 | 0.372; −0.556 |
| $R[F^2 > 4\sigma(F^2)]$ | 0.0868 | 0.0276 |
| w$R(F^2)$ | 0.2560 | 0.0664 |
| Goodness-of-fit, $S$ | 1.092 | 1.066 |

**Table S3.** Parameters of the supramolecular interactions and corresponding symmetry operators present in the prepared compounds.

| **Cocrystal** | **D···A** | ***d* / Å** | **∠ (X–D···A) / °** | **Symmetry Operator** |
|---|---|---|---|---|
| $CuCl_2$(**aap**)$_2$ | C8–H8C···O1 | 3.61(2) | 168.0 | *x, y, z* |
| | N1–H2N···Cl1 | 3.466(8) | 171(9) | *x, y, z* |
| [$CuCl_2$(**aap**)$_2$](**14tfib**) | I1···O1 | 2.989(2) | 172.6(1) | −*x*, −*y*, 1−*z* |
| | N1–H1A···Cl1 | 3.474(3) | 165(4) | *x, y, z* |

**Table S4.** Atom list and symmetry codes of symmetry equivalent atoms (marked with an ' symbol) in the prepared compounds.

| **$CuCl_2(aap)_2$** | | | | **$[CuCl_2(aap)_2](14tfib)$** | | | |
|---|---|---|---|---|---|---|---|
| **Atom** | **Symmetry Operator** | **Atom** | **Symmetry Operator** | **Atom** | **Symmetry Operator** | **Atom** | **Symmetry Operator** |
| C1 | *x, y, z* | H5 | *x, y, z* | C1 | *x, y, z* | F1' | −*x*, −*y*, 1−*z* |
| C2 | *x, y, z* | H6 | *x, y, z* | C2 | *x, y, z* | F2' | −*x*, −*y*, 1−*z* |
| C3 | *x, y, z* | H8A | *x, y, z* | C3 | *x, y, z* | I1 | *x, y, z* |
| C4 | *x, y, z* | H8B | *x, y, z* | C4 | *x, y, z* | I1' | −*x*, −*y*, 1−*z* |
| C5 | *x, y, z* | H8C | *x, y, z* | C5 | *x, y, z* | N1 | *x, y, z* |

| | | | | | | | |
|---|---|---|---|---|---|---|---|
| C6 | *x*, *y*, *z* | H1N' | −*x*, 1−*y*, −*z* | C6 | *x*, *y*, *z* | N1' | 1−*x*, −*y*, −*z* |
| C7 | *x*, *y*, *z* | H2N' | −*x*, 1−*y*, −*z* | C7 | *x*, *y*, *z* | O1 | *x*, *y*, *z* |
| C8 | *x*, *y*, *z* | H2' | −*x*, 1−*y*, −*z* | C8 | *x*, *y*, *z* | O1' | 1−*x*, −*y*, −*z* |
| C1' | −*x*, 1−*y*, −*z* | H3' | −*x*, 1−*y*, −*z* | C9 | *x*, *y*, *z* | H1A | *x*, *y*, *z* |
| C2' | −*x*, 1−*y*, −*z* | H5' | −*x*, 1−*y*, −*z* | C10 | *x*, *y*, *z* | H1B | *x*, *y*, *z* |
| C3' | −*x*, 1−*y*, −*z* | H6' | −*x*, 1−*y*, −*z* | C11 | *x*, *y*, *z* | H2 | *x*, *y*, *z* |
| C4' | −*x*, 1−*y*, −*z* | H8A' | −*x*, 1−*y*, −*z* | C1' | 1−*x*, −*y*, −*z* | H3 | *x*, *y*, *z* |
| C5' | −*x*, 1−*y*, −*z* | H8B' | −*x*, 1−*y*, −*z* | C2' | 1−*x*, −*y*, −*z* | H5 | *x*, *y*, *z* |
| C6' | −*x*, 1−*y*, −*z* | H8C' | −*x*, 1−*y*, −*z* | C3' | 1−*x*, −*y*, −*z* | H6 | *x*, *y*, *z* |
| C7' | −*x*, 1−*y*, −*z* | | | C4' | 1−*x*, −*y*, −*z* | H8A | *x*, *y*, *z* |
| C8' | −*x*, 1−*y*, −*z* | | | C5' | 1−*x*, −*y*, −*z* | H8B | *x*, *y*, *z* |
| Cu1 | *x*, *y*, *z* | | | C6' | 1−*x*, −*y*, −*z* | H8C | *x*, *y*, *z* |
| Cl1 | *x*, *y*, *z* | | | C7' | 1−*x*, −*y*, −*z* | H1A' | 1−*x*, −*y*, −*z* |
| Cl1' | −*x*, 1−*y*, −*z* | | | C8' | 1−*x*, −*y*, −*z* | H1B' | 1−*x*, −*y*, −*z* |
| N1 | *x*, *y*, *z* | | | C9' | −*x*, −*y*, 1−*z* | H2' | 1−*x*, −*y*, −*z* |
| N1' | −*x*, 1−*y*, −*z* | | | C10' | −*x*, −*y*, 1−*z* | H3' | 1−*x*, −*y*, −*z* |
| O1 | *x*, *y*, *z* | | | C11' | −*x*, −*y*, 1−*z* | H5' | 1−*x*, −*y*, −*z* |
| O1' | −*x*, 1−*y*, −*z* | | | Cu1 | *x*, *y*, *z* | H6' | 1−*x*, −*y*, −*z* |
| H1N | *x*, *y*, *z* | | | Cl1 | *x*, *y*, *z* | H8A' | 1−*x*, −*y*, −*z* |
| H2N | *x*, *y*, *z* | | | Cl1' | 1−*x*, −*y*, −*z* | H8B' | 1−*x*, −*y*, −*z* |
| H2 | *x*, *y*, *z* | | | F1 | *x*, *y*, *z* | H8C' | 1−*x*, −*y*, −*z* |
| H3 | *x*, *y*, *z* | | | F2 | *x*, *y*, *z* | | |
